# The role of circular RNAs in autoimmune diseases: Potential diagnostic biomarkers and therapeutic targets

**DOI:** 10.1096/fj.202401764R

**Published:** 2025-01-28

**Authors:** Xin’ai Li, Junhui Wang, Peng Wang, Shuo Qi, Jeremiah Amalraj, Jingwei Zhou, Zhiguo Ding

**Affiliations:** ^1^ Dongzhimen Hospital Beijing University of Chinese Medicine Beijing China; ^2^ Tongchuan City Thyroid Disease Prevention Center Tongchuan China; ^3^ Thyropathy Hospital, Sun Simiao Hospital Beijing University of Chinese Medicine Tongchuan China; ^4^ Lunenfeld‐Tanenbaum Research Institute Mount Sinai Hospital Toronto Ontario Canada; ^5^ The Key Laboratory of Cardiovascular Remodelling and Function Research, Chinese Ministry of Education and Chinese Ministry of Public Health, Department of Cardiology Qilu Hospital of Shandong University Jinan China; ^6^ Human Biology Program University of Toronto Toronto Ontario Canada; ^7^ The 1st Ward, Department of Nephrology and Endocrinology, Dongzhimen Hospital Beijing University of Chinese Medicine Beijing China

**Keywords:** autoimmune diseases, biomarker, circular RNA, diagnosis, treatment

## Abstract

With the emergence of high‐quality sequencing technologies, further research on transcriptomes has become possible. Circular RNA (circRNA), a novel type of endogenous RNA molecule with a covalently closed circular structure through “back‐splicing,” is reported to be widely present in eukaryotic cells and participates mainly in regulating gene and protein expression in various ways. It is becoming a research hotspot in the non‐coding RNA field. CircRNA shows close relation to several varieties of autoimmune diseases (AIDs) in both the physiological and pathological level and could potentially be used clinically in terms of diagnosis and treatment. Here, we focus on reviewing the importance of circRNA in various AIDs, with the aim of establishing new biomarkers and providing novel insights into understanding the role and functions of circRNA in AIDs. Specific signaling pathways of how circular RNAs are regulated in AIDs will also be illustrated in this review.

## Graphical abstract

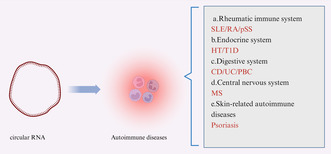

## 1 INTRODUCTION

**FIGURE 1:**
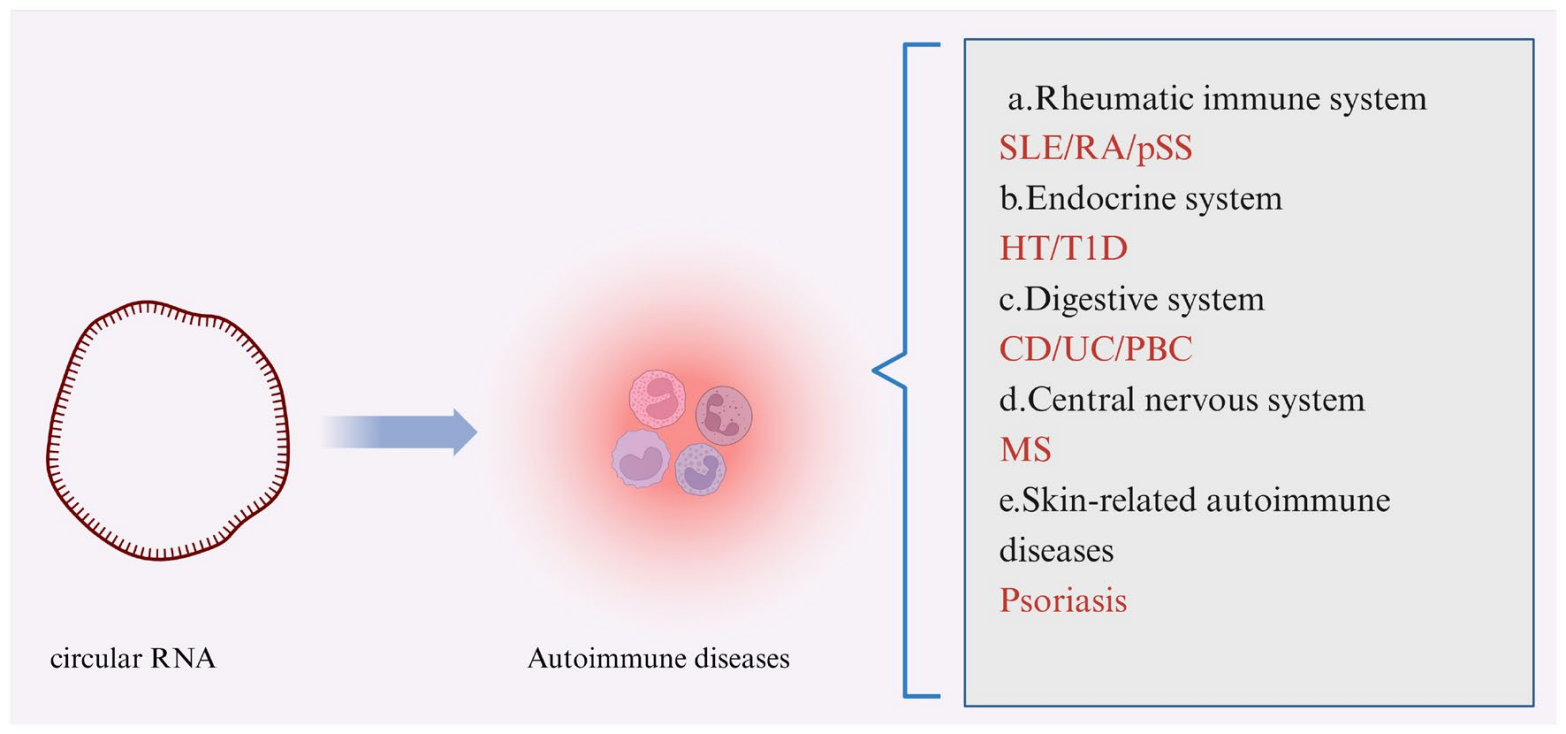
Advances in circRNA research in autoimmune disease.

AIDs are a group of chronic inflammatory diseases caused by autoimmune imbalances that affect multiple organ systems. A cohort study conducted in the United Kingdom screened 22 million individuals for 19 types of AIDs and found that 10.2% of the participants, with 63.9% being women, were diagnosed with AIDs. The prevalence of these diseases increased with age.^1^ The etiology of most of these conditions is not yet fully understood and there is a lack of effective treatments. This causes significant suffering for patients and places a heavy burden on the healthcare system. Therefore, the search for biomarkers and therapeutic targets for AIDs is of great importance for the advancement of treatment options for these diseases (Figure 1).

CircRNA, an endogenous circular non‐coding RNA family lacks the 5′ terminal cap and 3′ terminal poly(A) tail structure commonly found in mRNA. They are connected end to end by linking the upstream exon to the downstream exon with covalent bonds, which is called as “back‐splicing.”

CircRNA is generated through a process known as reverse splicing. In typical linear RNA splicing, introns are removed and exons are connected in a linear sequence. In contrast, during the production of circRNA, downstream splice donor sites are linked to upstream splice acceptor sites, resulting in a closed‐loop structure.^2^ Its typical functions include primarily acting as miRNA sponges, interacting with RNA‐binding proteins, regulating gene transcription, facilitating protein translation, etc.^3^, ^4^ CircRNAs are prevalent in eukaryotic organisms, including animals, plants, and fungi. They exhibit specific expression patterns across various tissues and cell types, and their expression levels fluctuate during developmental processes, disease states, and environmental stresses.^5^ In 1976, Sanger et al. first observed circRNA under an electron microscope when studying viroid RNA.^6^ However, it has often been regarded as an alternative splicing “byproduct” without function since its discovery.^7^ Advancements in high‐quality sequencing technology has led to the identification of an increasing number of circRNAs across diverse organisms. This discovery has illuminated their crucial role in biological functions and has expanded our comprehension of these complex molecular entities.^8^ For instance, Zhang et al. discovered that circLIFR‐007 decreased liver metastasis by facilitating the nuclear export of hnRNPA1 and promoting YAP phosphorylation in breast cancer.^9^ In recent years, numerous investigations have demonstrated notable participation of circRNAs in both transcriptional and translational modulations. Recent studies have demonstrated that circRNAs play a significant role in the onset and progression of various AIDs. These molecules may serve as sensitive and accurate diagnostic markers for AIDs. Furthermore, targeting circRNAs that are crucial in regulatory processes may enhance the therapeutic effects of treatments for AIDs and improve patient prognosis. Among them, the involvement in the initiation and progression of diverse AIDs has also been validated.^10^ This review will try to comprehensively outline the characteristics of circRNAs and their possible connections with AIDs. By unveiling the pivotal roles of circRNAs in AIDs, this review will bring about new conceptions of disease‐related biomarkers and promote new ideas of therapeutic strategy of AIDs.

To ensure consistency and clarity throughout the manuscript, we have all abbreviations delineating in Table 1 in this review for reference.

**TABLE 1:** Abbreviations and full names.

Abbreviations	Full name
circRNA	Circular RNA
AIDs	Autoimmune diseases
pre‐mRNA	MRNA precursors
DDX39A	DEAD‐box 39A
DDX39B	DEAD‐box 39B
RBPs	RNA‐binding proteins
miRNA	MicroRNAs
m6A	N6‐methyladenosine
DNA	Deoxyribonucleic acid
PKR	Protein kinase R
NF90	Nuclear factor 90
NF110	Nuclear factor 110
SLE	Systemic lupus erythematosus
PBMC	Peripheral blood mononuclear cell
HC	Healthy controls
ROC	Receiver operating characteristic
RT‐PCR	Reverse transcription‐polymerase chain reaction
AUC	Area under the curve
SLEDAI	SLE activity index
RA	Rheumatoid arthritis
PKBα	Protein kinase B α
CIA	Collagen‐induced arthritis
SJC	Swollen joint count
ACPA	Anti‐citrulline protein antibody
RF	Rheumatoid factor
TJC	Tender joint count
ATF2	Activating transcription factor 2
HAQ	Health assessment questionnaire
ESR	Erythrocyte sedimentation rate
DAS28	Disease activity score
CRP	C‐reactive protein
pSS	Primary Sjögren's syndrome
HT	Hashimoto's thyroiditis
T1DM	Type 1 diabetes mellitus
HuR	Human antigen R
IKK‐NF‐κB	iκB kinase‐nuclear factor‐κappa B
IBD	Inflammatory bowel diseases
CD	Crohn's disease
UC	Ulcerative colitis
CDAI	CD activity index
TNFα	Tumor necrosis factor‐α
IFN‐γ	Interferon‐γ
IL‐10	Interleukin‐10
PBC	Primary biliary cholangitis
3′‐UTR	3′‐untranslated region
SFRP2	Secreted frizzled‐related protein 2
TGF‐β	Transforming growth factor‐β
MS	Multiple sclerosis
RR‐MS	Relapsing remitting multiple sclerosis
MREs	MicroRNA response elements
IL‐17A	Interleukin‐17A
IL‐17 RA	IL‐17 receptor A
SARS‐CoV‐2	Severe acute respiratory syndrome coronavirus 2

## 2 SYNTHESIS, DEGRADATION, MECHANISM OF ACTION, AND PRIMARY FUNCTIONS OF circRNA

### 2.1 Bio‐synthesis and degradation of circRNA

**FIGURE 2:**
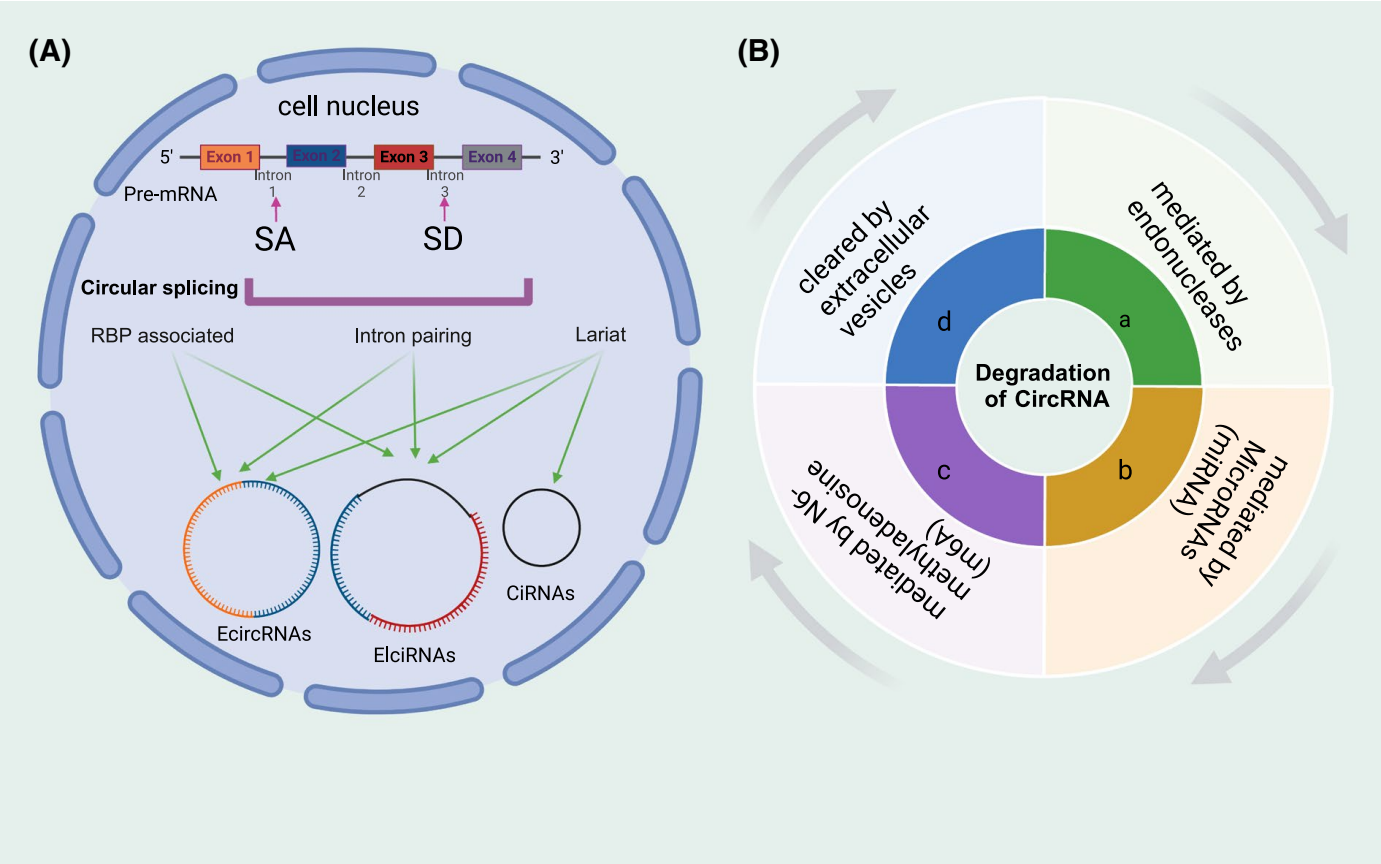
Synthesis and degradation of CircRNA (A) synthesis of circRNA; (B) degradation of CircRNA. SA, splice acceptor; SD, splice donor.

Diverging from linear RNA molecules, circRNA is generated through unconventional reverse splicing of particular mRNA precursors (pre‐mRNA). Classification of circRNA is based on distinct pre‐mRNA types, resulting in 4 categories: exon, intronic, exon–intron, and mitochondrial‐encoded circRNAs^11^ (Figure 2A). Proteins including RNA helicase DEAD‐box 39A (DDX39A) and RNA helicase DEAD‐box 39B (DDX39B) serve pivotal functions in the formation of these circular structures as they travel from the nucleus to the cytoplasm. Shorter circRNAs are transported by DDX39A, while longer circRNA transport is mediated by DDX39B.^12^ Different circRNAs have different linking patterns and their circularization can be achieved in approximately 3 ways: intron/lariat, RNA‐binding proteins (RBPs)‐mediated, and exon skipping.^13^ Due to its distinctive circular structure, conventional RNA degradation pathways are ineffective for the elimination of these circular RNA products, although their precise degradation mechanisms still remain unclear. The current proposed degradation models can be categorized into the following 4 methods: (a) mediated by endonucleases; (b) mediated by microRNAs (miRNAs); (c) mediated by N6‐methyladenosine (m6A); and (d) cleared by extracellular vesicles^13^ (Figure 2B).

### 2.2 Characteristics and biological functions of circRNA

**FIGURE 3:**
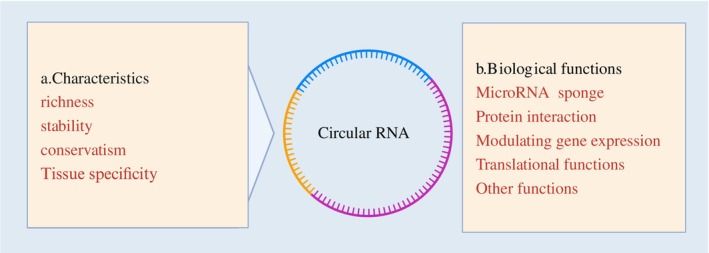
Characteristics and biological functions of circRNA.

CircRNA has the following characteristics: (a) richness: abundantly expressed in tissues, cells, cell components, and body fluids, with a wide variety and abundant content^14^; (b) stability: the unique circular structure of circRNA renders it less susceptible to degradation by extracellular nucleases, resulting in a unique covalently bound closed circular structure with a longer half‐life^15^; (c) conservatism: circRNA exhibits high conservation among different species, with some human circRNAs having corresponding homologous sequences in the genomes of other species^8^; and (d) tissue specificity: circRNAs are highly concentrated in the CNS and exhibit high specificity.^7^ The characteristics of circRNA make it a promising candidate as an effective biomarker for diseases. CircRNA expression exhibits tissue and cellular specificity, playing a functional role in physiological processes by regulating cell proliferation, autophagy, immunity, and development.^8^, ^16^ CircRNA exerts crucial functions in various physiological systems, including the circulatory/digestive/endocrine/respiratory/nervous/reproductive systems. An increasing number of researchers indicate that under pathological conditions, circRNA plays a crucial role in molecular level modulation, influencing the onset and progression of diseases through various mechanisms. These mechanisms encompass miRNA sponging, protein interaction, gene expression modulation, as well as other translational functions^14^, ^17^, ^18^, ^19^ (Figure 3).

### 2.3 The association of circular RNA with AIDs

**FIGURE 4:**
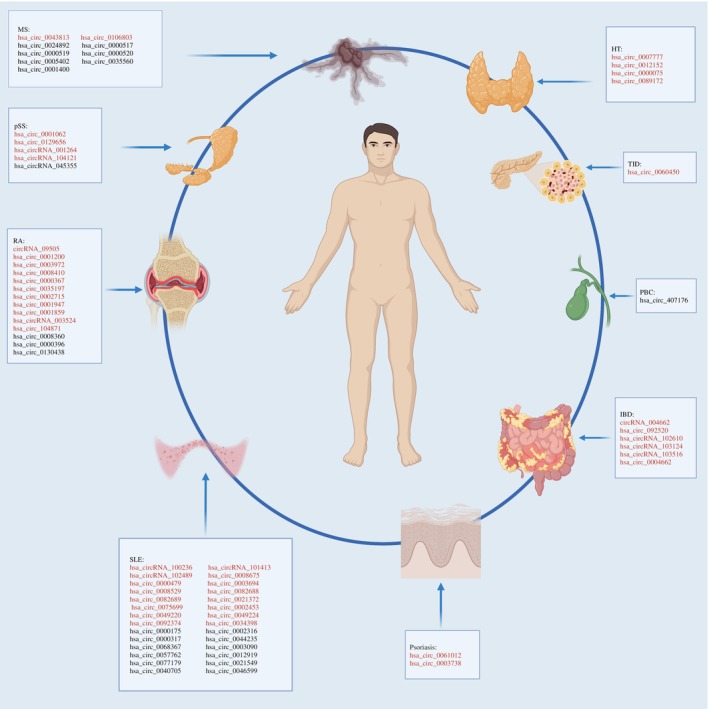
Disparities in circRNA expression in autoimmune diseases (Red font indicates up‐regulated circRNAs in disease, while black font indicates down‐regulated circRNAs in diseases).

Under normal circumstances, the body can recognize “self‐antigens” and typically does not elicit an immune response or elicits a small subdued response to its own components. In some cases, this state of self‐tolerance is disrupted, and the body's immune system produces an immune response against its own components, thereby producing autoantibodies or autoreactive T lymphocytes, resulting in AIDs.^20^ Existing research has illustrated that circRNA potentially facilitates immune responses and influences the development of AIDs. In the 1980s, scholars believed that cerebellar degeneration‐related protease I (a circRNA found in the human body) plays an important role in AIDs. It not only suppresses its own immunity and promotes inflammatory reactions but also stimulates the body to produce deoxyribonucleic acid (DNA) antibodies, thereby exacerbating the severity of the AID.^21^, ^22^ B cells have the capacity to differentiate into plasma cells, which in turn generate specific antibodies. Contemporary research suggests a close association between circRNA and the diverse transitions experienced by B cells in reaction to an immune stimulation. These studies highlight circRNA's contribution to crucial gene regulation in these processes.^23^ Agirre et al. identified 1356 circRNAs with notable expression within plasma cells.^23^ The circRNAs exhibiting higher expression levels in plasma cells were found to originate from Ig genes. In addition, they found a negative correlation between circRNA expression and the genes associated with cell proliferation, such as MKi67 and PCNA, in bone marrow and tonsil plasma cells. This indicates that circRNA expression potentially represents an unexplored regulatory mechanism in Ig site rearrangement within humoral immunity. It has been demonstrated that hsa_circ_0000652 is up‐regulated in patients with atherosclerosis, potentially acting as a sponge for hsa‐miR‐1179, which serves as a pro‐inflammatory factor in macrophages and a positive regulator of the OX40/OX40L pathway.^24^ It has also been demonstrated that elevated levels of hsa_circ_0090364 in patients with Graves’ disease is positively correlated with thyrotropin receptor antibodies. Further analysis indicates that hsa_circ_0090364 may regulate the JAK–STAT pathway through the hsa‐miR‐378a‐3p/IL‐6ST/IL21R axis, thereby promoting cell growth.^25^ Zhang et al. demonstrated that hsa_circRNA_405498 and hsa_circRNA_100033 are promising novel biomarkers for the differential diagnosis of T1D.^26^ Each rearranged Ig gene may give rise to produce a unique Ig‐derived circRNA, potentially elucidating the functional significance of circRNA in plasma cells. Previous research has indicated that endogenous circRNAs can easily form RNA duplexes spanning 16 to 26 base pairs in length.^27^, ^28^ The double‐stranded RNAs, along with the adenovirus small noncoding VAI RNA, function as a dsRNA‐activated protein kinase R (PKR) suppressor which is an innate immunity‐associated component.^29^ Upon viral infection or exposure to poly (I:C), circRNAs undergo global degradation, causing the activation of PKR within the innate immune response's early phase. Moreover, circRNAs exhibit close associations with immune factors, nuclear factor 90(NF90)/(nuclear factor 110, NF110) within the context of innate immunity. NF90 and NF110, which are splicing variants derived from the ILF3 gene, constitute dsRNA‐binding proteins structured by inverted repeat elements situated in the introns surrounding their respective splicing sites. These aforementioned variants stabilize RNA hairpin structures, which facilitate reverse splicing. Following viral invasion, NF90/NF110 undergo nuclear‐to‐cytoplasmic transport, resulting in a global reduction in circRNA levels. Furthermore, these proteins can dissociate from the complex and bind to viral mRNA, thereby inhibiting viral replication.^30^, ^31^ In immune aging, a noteworthy increase in CD8+ CD28− T cells has been observed. Researchers propose that hsa_circ_100783 in aged human CD8+ T cells potentially functions as an innovative marker for the aging of CD8+ T cells, which is correlated with CD28.^32^ The findings above offer compelling evidence that circRNAs play pivotal roles as regulators of immunity. Several studies have also reported associations between circRNA and autoimmune diseases. (Figure 4) (Table 2).

**TABLE 2:** Expression and function of circRNA in AIDs.

circRNA name	Disease name	Host gene	Expression pattern	Clinical significance	Detect object tissues/cells	Detection technology chip sequencing/PCR	PMID
hsa_circRNA_100236	Systemic lupus erythematosus	Unknown	Up‐regulated	Novel biomarkers for the diagnosis of SLE	PBMC	qRT‐PCR	33750387
hsa_circRNA_101413	Systemic lupus erythematosus	Unknown	Up‐regulated				33750387
hsa_circRNA_102489	Systemic lupus erythematosus	Unknown	Up‐regulated				33750387
hsa_circ_0008675	Systemic lupus erythematosus	ZNFX1	Up‐regulated	Potential biomarkers for the diagnosis and treatment of systemic lupus erythematosus with renal involvement (SLE+RI)	Peripheral blood	qRT‐PCR	32940208
hsa_circ_0000175	Systemic lupus erythematosus	ELK4	Down‐regulated	As a potential biomarker for SLE diagnosis	PBMC	qRT‐PCR	32093518
hsa_circ_0002316	Systemic lupus erythematosus	OMA1	Down‐regulated				32093518
hsa_circ_0000317	Systemic lupus erythematosus	AHNAK	Down‐regulated				32093518
hsa_circ_0000479	Systemic lupus erythematosus	EPSTI1	Up‐regulated				32093518
hsa_circ_0003694	Systemic lupus erythematosus	SMAD2	Up‐regulated				32093518
hsa_circ_0008529	Systemic lupus erythematosus	ACTR2	Up‐regulated				32093518
hsa_circ_0082688	Systemic lupus erythematosus	PARP12	Up‐regulated				32093518
hsa_circ_0082689	Systemic lupus erythematosus	PARP12	Up‐regulated				32093518
hsa_circ_0044235	Systemic lupus erythematosus	CDC27	Down‐regulated	It has potential as a diagnostic biomarker for SLE	PBMC	RT‐qPCR	31432107
hsa_circ_0068367	Systemic lupus erythematosus	VPS8	Down‐regulated				31432107
hsa_circ_0021372	Systemic lupus erythematosus	RPS13	Up‐regulated	It can be used as a biomarker for SLE diagnosis	Whole blood	RT‐PCR	30628013
hsa_circ_0003090	Systemic lupus erythematosus	PLAUR	Down‐regulated				30628013
hsa_circ_0057762	Systemic lupus erythematosus	STRADB	Down‐regulated				30628013
hsa_circ_0075699	Systemic lupus erythematosus	FAM8A1	Up‐regulated				30628013
hsa_circ_0012919	Systemic lupus erythematosus	GNG12	Down‐regulated	Markers for diagnosing SLE	CD4 T cell	RT‐qPCR	30237316
hsa_circ_0002453	Systemic lupus erythematosus	RAD18	Up‐regulated	Diagnostic markers for patients with lupus nephritis	Plasma	qRT‐PCR	30172209
hsa_circ_0077179	Systemic lupus erythematosus	IBTK	Down‐regulated	It can be used as a biomarker and therapeutic target for SLE	PBMC	RT‐qPCR	29884225
hsa_circ_0049220	Systemic lupus erythematosus	DNMT1	Up‐regulated	It may be related to the potential pathogenesis of SLE and has potential diagnostic value for SLE	PBMC	RT‐qPCR	29606700
hsa_circ_0049224	Systemic lupus erythematosus	DNMT1	Up‐regulated				29606700
hsa_circ_0092374	Systemic lupus erythematosus	GADD45A	Up‐regulated	Biomarkers of SLE	Plasma	qRT‐qPCR	29360436
hsa_circ_0021549	Systemic lupus erythematosus	MPPED2	Down‐regulated				29360436
hsa_circ_0034398	Systemic lupus erythematosus	C15orf41	Up‐regulated				29360436
hsa_circ_0040705	Systemic lupus erythematosus	USP10	Down‐regulated				29360436
hsa_circ_0046599	Systemic lupus erythematosus	B3GNTL1	Down‐regulated				29360436
circRNA_09505	Rheumatoid arthritis	Unknown	Up‐regulated	As a target for RA immunotherapy	PBMC	PCR	33028811
hsa_circ_0001200	Rheumatoid arthritis	PTTG1IP	Up‐regulated	Potential biomarkers for the diagnosis of RA	PBMC	RT‐qPCR	32191279
hsa_circ_0008360	Rheumatoid arthritis	XPNPEP3	Down‐regulated				32191279
hsa_circ_0003972	Rheumatoid arthritis	FAM120A	Up‐regulated				32191279
hsa_circ_0008410	Rheumatoid arthritis	PGS1	Up‐regulated	Markers of diagnosis and disease progression	PBMC	RT‐qPCR	32124964
hsa_circ_0000367	Rheumatoid arthritis	SIAE	Up‐regulated	A potential biomarker for patients with RA and maybe associated with disease activity.	Peripheral Blood	qRT‐PCR	31772684
hsa_circ_0035197	Rheumatoid arthritis	ATP8B4	Up‐regulated				31772684
hsa_circ_0002715	Rheumatoid arthritis	PCNT	Up‐regulated				31772684
hsa_circ_0001947/hsa_circ_000510	Rheumatoid arthritis	AFF2	Up‐regulated				31772684
hsa_circ_0000396	Rheumatoid arthritis	SLC38A1	Down‐regulated	Biomarkers for the diagnosis of RA	PBMC	qT‐PCR	31596149
hsa_circ_0130438	Rheumatoid arthritis	KPNA5	Down‐regulated				31596149
hsa_circ_0001859/hsa_circ_001783	Rheumatoid arthritis	TCONS_l2_00028722	Up‐regulated	Involved in the process of chronic inflammatory diseases in synovial tissue	PBMC	qT‐PCR	29577053
hsa_circRNA_003524	Rheumatoid arthritis	FAM168B	Up‐regulated	RA diagnostic markers	PBMC	qT‐PCR	28618429
hsa_circ_104871	Rheumatoid arthritis	SUSD1	Up‐regulated				28618429
hsa_circ_0001062	Primary Sjogren's Syndrome	ZC3H6	Up‐regulated	Diagnostic markers of PSS	PBMC	PCR	32250392
hsa_circ_0129656	Primary Sjogren's Syndrome	IQGAP2	Up‐regulated				32250392
hsa_circRNA_001264	Primary Sjogren's Syndrome	Unknown	Up‐regulated	Biomarkers of pSS	PBMC	PCR	31420809
hsa_circRNA_045355	Primary Sjogren's syndrome	Unknown	Down‐regulated				31420809
hsa_circRNA_104121	Primary Sjogren's syndrome	Unknown	Up‐regulated				31420809
hsa_circ_0007777	Hashimoto's thyroiditis	KHDRBS1	Up‐regulated	Potential diagnostic biomarker of HT and may play a crucial role in the pathogenesis of HT via sponging miR‐125a‐3p	PBMC	RT‐qPCR	31207490
hsa_circ_0012152	Hashimoto's thyroiditis	RNF220	Up‐regulated				31207490
hsa_circ_0000075	Hashimoto's thyroiditis	FGGY	Up‐regulated				31207490
hsa_circ_0089172	Hashimoto's thyroiditis	NUP214	Up‐regulated				31207490
hsa_circ_0060450	Type1 diabetes mellitus	MYBL2	Up‐regulated	Inhibition of macrophage‐mediated inflammation is a promising therapeutic molecule in T1DM therapy	PBMC	RT‐qPCR	33133095
circRNA_004662	Crohn disease	Unknown	Up‐regulated	Biomarkers for the diagnosis of CD	PBMC	RT‐PCR	31261517
hsa_circ_092520	Crohn disease	Unknown	Up‐regulated				31261517
hsa_circRNA_102610	Crohn disease	Unknown	Up‐regulated				31261517
hsa_circRNA_103124	Crohn disease	Unknown	Up‐regulated	Diagnose CDS and distinguish between CDS and new candidates for UC			31261517
hsa_circRNA_103516	Inflammatory bowel disease	FNDC3B	Up‐regulated	Biomarkers for the diagnosis of IBD	PBMC	RT‐PCR	31749597
hsa_circ_0004662	Ulcerative colitis	SOD2	Up‐regulated	Biomarkers for UC diagnosis	Tissues	PCR	31624381
hsa_circ_0052372	Primary biliary cholangitis	TRIM28	Unknown	Biomarkers for diagnosis of PBC	Plasma	qRT‐PCR	29183005
hsa_circ_407176	Primary biliary cholangitis	ZCCHC7	Down‐regulated				29183005
hsa_circ_0043813	Multiple sclerosis	STAT3	Up‐regulated	MS diagnostic markers	PBMC	RT‐PCR	30619471
hsa_circ_0024892	Multiple sclerosis	SNX19	Down‐regulated	As a new biomarker for multiple sclerosis	Peripheral blood	qPCR	28651352
hsa_circ_0000517	Multiple sclerosis	RPPH1	Down‐regulated				28651352
hsa_circ_0000519	Multiple sclerosis	RPPH1	Down‐regulated				28651352
hsa_circ_0000520/hsa_circ_001846	Multiple sclerosis	RPPH1	Down‐regulated				28651352
hsa_circ_0005402/hsa_circ_0000518/hsa_circ_000167	Multiple sclerosis	ANXA2	Down‐regulated				28651352
hsa_circ_0035560/hsa_circ_0005402/hsa_circRNA_101539/hsa_circRNA_101541	Multiple sclerosis	ANXA2	Down‐regulated				28651352
hsa_circ_0001400	Multiple sclerosis	RELL1	Down‐regulated				28651352
hsa_circ_0106803	Multiple sclerosis	GSDMB	Up‐regulated		PBMC	RT‐PCR	28272342
hsa_circ_0061012	Psoriasis	SLMO2‐ATP5E	Up‐regulated	Biomarkers for psoriasis	Tissues	PCR	30278433
hsa_circ_0003738	Psoriasis	LRRC16A	Up‐regulated	The miR‐562/IL‐17 RA and miR‐490‐5p/IFNGR2 (IFN‐gamma receptor 2) axes reduce the inhibitory function of Tregs in psoriasis	PBMC	PCR	32871353

### 2.4 The role of circular RNA in AIDs

#### 2.4.1 CircRNA and systemic lupus erythematosus (SLE)

CircRNA has been linked to multiple AIDs affecting the rheumatic immune system. Systemic lupus erythematosus (SLE) is a multifaceted systemic autoimmune disease.^33^ This condition presents a myriad of renal, dermatological, neuropsychiatric, and cardiovascular symptoms.^34^ Predominantly affecting young women, SLE requires a complex clinical course with prolonged and recurrent features commonly occurring after treatment, yet its exact pathogenesis remains elusive.^27^ Recently, an expanding body of researchers has emphasized the involvement of circRNAs in gene transcriptional regulation, implicating them in the complex landscape of SLE development and progression, and other AIDs.^10^ Zheng et al. determined both hsa_circRNA_100236 and _101413 expression in peripheral blood mononuclear cells (PBMCs) from healthy individuals and SLE subjects.^35^ The microarray findings were corroborated through qRT‐PCR validation. Exploring the SLE clinical‐pathological correlations, the researchers identified the associations between particular circRNAs and certain distinct manifestations: hsa_circRNA_101413, hsa_circRNA_102489, and hsa_circRNA_100236 were linked to IgG, thrombocytopenia, and anti‐dsDNA, respectively. This implies the potential use of circRNAs as diagnostic markers. Luo et al.^36^ conducted an extensive analysis and comparison of peripheral blood samples from three categories: individuals with renal involvement (SLE+RI); without renal involvement (SLE‐RI); and healthy controls (HC). These studies validated the potential of role of some circRNAs within SLE pathophysiology, paving the way for potentially developing diagnostic and therapeutic advancements in the field. Utilizing qRT‐PCR, this research team also confirmed that hsa_circ_0008675 expression was notably elevated in SLE+RI compared to SLE‐RI and HC. Significantly, hsa_circ_0008675 exhibited an association with certain treatment outcomes. Receiver operating characteristic (ROC) curve analysis indicated a higher diagnostic value of hsa_circ_0008675 in newly diagnosed SLE+RI individuals compared to the control cohort (newly diagnosed SLE‐RI individuals and HC). Thus, hsa_circ_0008675 could work as a potential biomarker for evaluation on the diagnosis of SLE and the treatment effectiveness in individuals with renal injury. In a genomic comparison study conducted by Luo et al., studying PBMCs from 23 newly diagnosed SLE individuals and 23 healthy controls, reverse transcription‐polymerase chain reactions (RT‐PCR) revealed significantly elevated hsa_circ_0000479, _0082688, _0082689, _0008529, and _0003694 expressions in SLE individuals compared to HC cohort.^36^ Conversely, hsa_circ_0002316, _0000317, and _0000175 expressions within SLE were significantly reduced compared to the HC cohort. Moreover, Luo et al. examined PBMCs from 30 individuals with SLE and 20 healthy controls.^37^ They observed a significant decrease in the expressions of hsa_circ_0044235 and _0068367. Consistent results obtained through qRT‐PCR suggested that the reduced hsa_circ_0044235 and _0068367 expressions have the potential to be used as diagnostic markers for SLE. Li et al. explored circRNA levels in SLE children, revealing a decreased level of expression of hsa_circ_0057762 and _0003090.^38^ Additionally, hsa_circ_0021372 and _0075699 in children were found to be associated with elevated C3 and C4 levels compared to those observed in healthy individuals. Further ROC curve analysis demonstrated that hsa_circ_0057762 [area under the curve (AUC) 0.804, 95% CI 0.607–1.0, *p* = .02] and _0003090 (AUC 0.848, 95% CI 0.688–1.0, *p* = .008) levels effectively distinguish SLE individuals from HC, suggesting their potential as diagnostic markers for SLE. In another study by Zhang et al., among SLE individuals and healthy controls, CD4+ T cells were isolated and a circRNA microarray analysis was used to screen for a circRNA candidate in these CD4+ T cells.^39^ Following transfection with hsa_circ_0012919‐targeted small interfering RNA (siRNA), the levels of DNA methyltransferase (DNMT1), CD11a, and CD70 along with CD11a and CD70 methylation levels, were evaluated. Using bioinformatics, the authors employed a network analysis of hsa_circ_0012919. A luciferase reporter gene assay and fluorescence in situ hybridization assay was used to identify miRNAs interacting with hsa_circ_0012919. Results showed a reduced expression of hsa_circ_0012919 in SLE individuals compared to HC. These results further validated the connection of circRNA to SLE. Hsa_circ_0012919 has been identified as a biomarker and operates as a competing endogenous RNA for miR‐125a‐3p. Yang et al.^40^ performed a microarray screening for circRNA changes in individuals with lupus nephritis plasma, SLE patients without lupus nephritis, and HC. RT‐PCR verification identified notably elevated levels of plasma circRNA_002453 in those with lupus nephritis, with no significant correlation to disease activity. Zhang et al. utilized RT‐PCR to measure the expressions of hsa_circ_0049224, hsa_circ_0049220, and DNMT1 in the PBMCs of 18 patients and 10 healthy controls.^41^ The results indicated that hsa_circ_0049224 and _0049220 expressions in the HC cohort notably surpassed both inactive and active SLE individuals. Moreover, DNMT1 expression was positively connected with the expressions of hsa_circ_0049224 and _0049220. Furthermore, a negative correlation was noted between the SLE activity index (SLEDAI) and the expressions of hsa_circ_0049224 and _0049220. These findings suggested that hsa_circ_0049224 and _0049220 might serve as potential contributors to SLE pathogenesis, giving it potential to be used in clinical applications. Li et al. showed the increased expression of hsa_circ_0092374 and _0034398, alongside the decreased expression of hsa_circ_0021549, _0040705, and _0046599 within SLE patients.^42^ These studies laid the groundwork for circRNAs' future development as an innovative non‐invasive SLE biomarker. As high‐throughput and microarray technologies rapidly advance, more researchers attest the importance of circRNAs in the onset and progression of SLE. The aforementioned studies indicate that circRNA may be involved in the activation of immune cells, the inflammatory response, and the generation of autoantibodies. In addition, circRNA may also be involved in the development of SLE. Nonetheless, further exploration is needed to provide more evidence to further verify that circRNAs could play a potential role in terms of SLE diagnosis and treatments in the future. The potential uses for circRNAs as markers encompasses many fields including diagnosis, treatment efficacy evaluation, and prognosis, providing new opportunities for investigating SLE pathogenesis and identifying potential therapeutic targets in a novel way.

#### 2.4.2 CircRNA and rheumatoid arthritis (RA)

RA, a chronic inflammatory joint disorder, having the potential to cause cartilage and bone damage, can possibly cause permanent disability. The incidence rate varies from 0.5% to 1%.^43^ As Yang et al. illustrated, circRNA_09505 showed the most notable increase in the PBMCs of RA patients.^44^ By acting as a miR‐6089 sponge and targeting protein kinase B α (PKBα) in vitro, it regulated inflammation in macrophages by inhibiting circRNA_09505 in macrophages resulting in a reduction in inflammation and joint damage in mice with collagen‐induced arthritis (CIA). Therefore, the circRNA_09505‐miR‐6089 ceRNA network serves as a promising target for both RA treatment and identification. Wen et al.^45^ conducted sequencing of miRNA and circRNAs in the PBMCs of RA patients and healthy controls. Through RT‐qPCR verification of differentially expressed circRNAs in age‐ and gender‐matched controls from RA patients, they found a decrease in hsa_circ_0008360 expression and an increase in hsa_circ_0001200 and _0003972 expressions, which was consistent with their sequencing results. These circRNAs may influence RA onset and progression and could potentially serve as RA biomarkers as well. Luo et al. analyzed 6 circRNAs in RA individuals and healthy controls using RT‐qPCR.^46^ The assessment of hsa_circ_0008410 in an independent cohort, including RA patients, SLE patients, ankylosing spondylitis (AS) patients, and healthy controls, revealed a notable increase in its expression among RA patients. The expression of hsa_circ_0008410 was associated with disease duration, tender joint count, platelet count, and platelet volume ratio, suggesting corresponding connection with RA activity and severity. Luo et al. found significantly higher expression levels of hsa_circ_0002715, _0001947, _0000367, and _0035197 in the peripheral blood of new‐onset RA compared to HC.^47^ Logistic regression models indicated that the combination of hsa_circ_0002715 and _0035197 provided the most optimal accuracy for diagnosis, with an AUC of 0.758 (sensitivity: 72.9%, specificity: 71.4%). Peripheral blood hsa_circ_0002715 level was associated with disease duration, swollen joint count (SJC), anti‐citrulline protein antibody (ACPA), rheumatoid factor (RF), and tender joint count (TJC). This suggests a potential indicative role of hsa_circ_0002715 and _0035197 combination with peripheral blood for new‐onset RA patients, linking directly to RA's disease activity. In a comprehensive study, Yang et al. utilized both RNA‐seq and microarray analyses, revealing higher expression of hsa_circ_0000396 and _0130438 within the RA cohort compared to the HC cohort. This dual approach strengthens the potential role of circRNAs from a diagnostic perspective.^48^ Li et al.'s silencing of hsa_circ_0001859 resulted in inhibited activating transcription factor 2 (ATF2) expression and reduced SW982 cells inflammation level.^49^ Significantly, hsa_circ_0001859 could compete with ATF2 for miR‐204/211, which indicates its significant involvement in chronic inflammatory processes in synovial tissue. Yang et al. conducted microarray screening for circRNA alterations in the PBMCs of RA patients and HC.^50^ Confirmation through qRT‐PCR in a larger cohort established the diagnostic significance of circRNA_104871 for RA, with an AUC of 0.833 (*p* < .001). CircRNA_003524 also demonstrated considerable diagnostic potential (AUC = 0.683, *p* = .020). Nevertheless, these circRNAs showed no notable connection with disease severity indicators, inclusive of health assessment questionnaire (HAQ), erythrocyte sedimentation rate (ESR), disease activity score (DAS28), and C‐reactive protein (CRP). The increased expression of circRNA_104871 and _003524 in RA patients' PBMCs may serve as potential RA diagnostic markers, independent of disease severity indicators. Therefore, circRNA may play a role in modulating inflammation and the immune response in RA and may become a novel target for diagnosis and treatment. Some changes in the expression levels correlate with the onset, progression, and gravity of the ailment, which may serve as biomarkers of diagnosis and prognosis. But further studies are needed to substantiate these findings and investigate the precise action mechanism of circRNAs in RA.

#### 2.4.3 CircRNA and primary Sjögren's syndrome (pSS)

**FIGURE 5:**
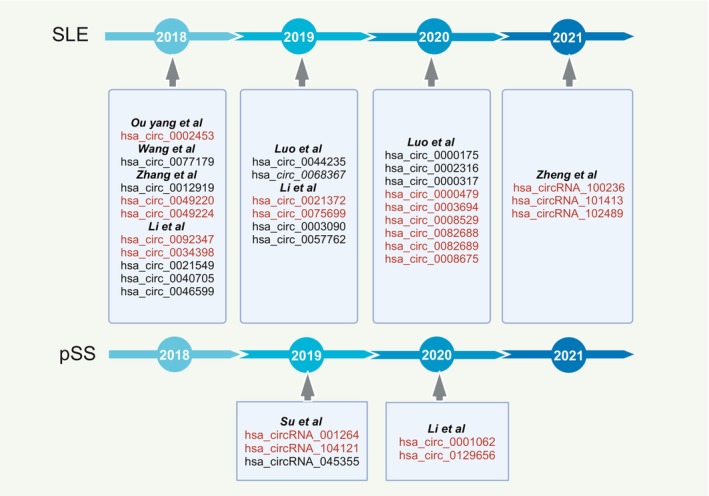
Role of circRNA in SLE and pSS (Red font indicates up‐regulated circRNAs in disease, while black font indicates down‐regulated circRNAs in diseases).

pSS, an inflammatory disorder, primarily affects the salivary and lacrimal glands. However, patients with pSS may also experience extra‐glandular complications such as tubulointerstitial nephritis and autoimmune hepatitis, attributed to autoantibody overexpression, resulting in hypergammaglobulinemia. The precise etiology remains unclear but akin to other autoimmune conditions, both genetic and environmental factors are implicated in its onset.^51^ Li et al. revealed circ‐IQGAP2 and circ‐ZC3H6 could probably function as non‐invasive pSS markers with circular RNA sequencing.^52^ Su et al. utilized microarray analysis to examine PBMCs circRNA expression from 5 pairs of pSS, SLE patients, and controls, validating differentially expressed circRNAs through qRT‐PCR.^53^ The study identified that hsa_circRNA_001264, _104121, and _045355 were closely associated with various laboratory parameters, disease activity indices, and clinical manifestations in pSS patients. The ROC analysis indicated the potential diagnostic capability of these 3 circRNAs. Using a microarray analysis of circRNA expression in PBMCs, it was found that hsa_circRNA_001264 and hsa_circRNA_104121 expressions were up‐regulated and hsa_circRNA_045355 expression was decreased in pSS patients compared with healthy individuals. Furthermore, early‐stage pSS individuals exhibited higher expression levels of hsa_circRNA_001264 and _104121, and lower expression of hsa_circRNA_045355 compared to those with advanced pSS. Notably, the dysregulation of these 3 circRNAs was correlated closely with the disease activity index. CircRNAs exert their regulatory functions by competitively binding and inhibiting specific miRNAs. In the case of hsa_circRNA_104121, it binds and inhibits miR‐203a‐3p and miR‐143‐3p. Hsa_circRNA_104121 is elevated in pSS individuals, while individuals with compromised oral health exhibit higher miR‐203a‐3p expression in saliva.^54^ Additionally, miR‐143‐3p expression in RA synovial tissue significantly exceeds that of control group. MiR‐143‐3p expression suppression inhibited cell proliferation, reduced inflammatory cytokine levels, and promoted cell apoptosis, highlighting circRNAs' aberrant expression patterns in these individuals.^55^ These above studies all suggested that circRNA is abnormally expressed in pSS, which may modulate inflammation and the immune response through competitive binding and inhibiting of specific miRNAs. Thus, they could be used as a potential diagnostic marker for pSS. The role of circRNA in SLE and pSS is shown in Figure 5.

## 3 CircRNA IS LINKED TO AUTOIMMUNE DISEASES OF THE ENDOCRINE SYSTEM

### 3.1 CircRNA and HT

Hashimoto's thyroiditis (HT), marked by the presence of thyroid‐specific autoantibodies, is a common autoimmune disorder. While the precise etiological mechanism remains elusive, HT was believed to involve intricate interactions among epigenetic, environmental, and genetic factors.^56^ It stands as a leading cause of hypothyroidism.^56^, ^57^, ^58^ In a study by *Si* et al., peripheral blood specimens from 5 HT patients and 5 healthy volunteers were analyzed utilizing Illumina HiSeq sequencer.^59^ The findings revealed elevated expression of hsa_circ_0007777, _0012152, _0000075, and _0089172 in HT patients. Notably, hsa_circ_0089172 expression exhibited up‐regulation, demonstrating a positive connection with thyroid peroxidase antibody levels within the serum. Bioinformatics tools identified 2 perfect miR‐125a‐3p‐matched binding sites within the hsa_circ_0089172 sequence. MiR‐125a‐3p expression was diminished in HT subjects and exhibited negative association with increased hsa_circ_0089172. Furthermore, hsa_circ_0089172 in vitro knockout could lead to miR‐125a‐3p expression augmentation. ROC analysis underscored the notable HT diagnostic efficiency of hsa_circ_0089172. In summary, the overall outcome proposes hsa_circ_0089172 as a promising candidate for a potential diagnostic biomarker of HT. Furthermore, it has a potential role in the pathogenesis of the condition, indicating that it possibly could act as a regulatory element by sequestering miR‐125a‐3p. In conclusion, hsa_circ_0089172 could serve as a potential diagnostic biomarker for HT and might be implicated in the pathogenesis of the disease by sponging miR‐125a‐3p.

### 3.2 CircRNA and T1DM

Type 1 diabetes mellitus (T1DM) stands as a chronic autoimmune disorder characterized by the immune‐mediated destruction of pancreatic β cells responsible for insulin production. The development of this condition is influenced by an intricate interaction of genetic and environmental factors.^60^ Referring from International Diabetes Federation Atlas, approximately 1.2 million children and adolescents were diagnosed within 2021.^61^ CircRNAs have served pivotally in diabetes‐related processes, including insulin synthesis, secretion, programmed apoptosis of pancreatic β cells, insulin resistance, and pancreatic inflammation. Kaur et al. identified 10 830 circRNAs in human pancreatic α, β, and exocrine cells.^62^ Most circRNAs were predominantly located in pancreatic α and β cells, showing selective expression patterns linked to blood glucose levels, insulin resistance, pancreatic inflammation, and cellular processes such as proliferation and apoptosis in diabetic individuals. In a study by Zhang et al., microarray analysis revealed a significant up‐regulation of circPPM1F parental gene protein phosphatase 1F (PPM1F) expression in PBMCs from individuals with T1DM.^63^ This finding suggests that circPPM1F plays a potential role in the regulatory mechanisms of T1DM. Subsequent research has revealed the complex mechanisms involving circPPM1F in T1DM. The circPPM1F molecule was found to competitively bind with human antigen R (HuR), thereby impeding PPM1F expression. The blockade counteracted the suppressive effect of PPM1F on the iκB kinase‐nuclear factor‐κappa B (IKK‐NF‐κB) pathway, fostering M1‐type macrophages and initiating pancreatic inflammation. Concurrently, the collaborative action of the eukaryotic translation initiator (EIF4A3) and the fused in sarcoma (FUS) RNA‐binding protein served pivotally in regulating circPPM1F generation, thereby ensuring circPPM1F sustained expression levels throughout T1DM progression. Yang et al. provided differential circRNA expression profiles obtained through microarray analysis from children with T1DM and age‐matched healthy controls.^64^ The top 10 circRNAs, exhibiting the highest fold differences, were subsequently validated using RT‐qPCR in clinical PBMC specimens from T1DM children and healthy controls, respectively. Among these, hsa_circ_0060450 emerged as detectable and significantly elevated circular RNA in the PBMCs of T1DM individuals compared to the control group. ROC analysis also further demonstrated that in PBMCs, hsa_circ_0060450 has the potential to work as a biomarker to distinguish T1DM versus normal subjects. Subsequent experiments elucidated that hsa_circ_0060450 could function by sequestering miR‐199a‐5p, liberating its target src homology 2 domain‐containing phosphatase 2 (SHP2) and suppress the I‐type interferon‐triggered janus kinase 1‐signal transducer and activator of transcription 1/3 (JAK‐STAT1/3) signaling pathway, which ultimately inhibits macrophage‐mediated inflammation. Collectively, this research underscores hsa_circ_0060450's pivotal role within T1DM, suggesting its promising role as a prospective therapeutic target for future T1DM treatments.

### 3.3 CircRNA is associated with autoimmune diseases of the digestive system

#### 3.3.1 CircRNA and IBD

Inflammatory bowel diseases (IBD), which include subtypes like Crohn's disease (CD) and ulcerative colitis (UC), are chronic relapsing gastrointestinal diseases that most commonly affect children, adolescents, and young adults.^65^ Extra‐intestinal complications may also be manifested. CD incidence is on the rise in both in adults and children.^66^ Increasing evidence indicates circRNAs are implicated in IBD and are considered promising markers for these diseases. Ye et al. conducted a study involving 90 CD patients, 90 with UC, 80 HC, and 35 patient controls (PC).^67^ Compared to HC and PC, circRNA_103516 expression was escalated in CD and UC patients (*p* < .05). The diagnostic potential of circRNA_103516 for CD and UC was demonstrated by an AUC of 0.790 and 0.687, respectively. Significantly, circRNA_103516 levels within active CD and UC cohorts surpassed those in remission cohorts (*p* = .027, *p* = .045). To CD, circRNA_103516 was positively associated with the ESR, CRP, CD activity index (CDAI), tumor necrosis factor‐α (TNFα), and interferon‐γ (IFN‐γ) (*p* < .001), and negatively correlated with interleukin‐10 (IL‐10) (*p* = .006). For UC, circRNA_103516 was related to CRP, ESR, Mayo score, TNFα (*p* < .001), IFN‐γ (*p* = .011), and IL‐10 (*p* = .002). Furthermore, has‐miR‐19b‐1‐5p exhibited a negative correlation with circRNA_103516 in CD, suggesting its potential as a promising candidate marker for IBD identification. CircRNA_103516 dysregulation is involved in IBD molecular mechanisms by which circRNA_103516 sponged for has‐miR‐19b‐1‐5p. Yin et al. analyzed PBMCs from 5 individuals with CD and 5 healthy controls via microarray analysis.^68^ The differential expression was confirmed by RT‐PCR, revealing 155 up‐regulated and 229 down‐regulated circRNAs. 4 circRNAs (092520, 102610, 004662, and 103124) were notably up‐regulated in CD in comparison to the healthy control group, as verified by RT‐PCR and qPCR. ROC analysis showed a diagnostic significance of these circRNAs in CD, with respective AUC values of 0.66, 0.78, 0.85, and 0.74, respectively. The up‐regulation of these four circRNAs in PBMCs may serve as potential CD diagnostic markers, with circRNA_004662 showing the most promise as new‐established candidate for distinguishing between CD and UC. Wang et al. selected five pairs of inflammatory colorectal mucosal tissues and normal mucosal tissues from individuals for circular RNA microarray detection.^69^ The circular hsa_circ_0004662 was significantly up‐regulated, as further verified by real‐time fluorescent qPCR in 30 cases of inflammatory colorectal mucosal tissues and normal mucosal tissues from UC individuals. The elevated hsa_circ_0004662 in UC was found to impair the intestinal epithelial barrier, suggesting its potential involvement in the development. CircRNA_103516 could be used as a diagnostic biomarker for IBD, whose dysregulation is implicated in the molecular mechanism of IBD through sponging with has‐miR‐19b‐1‐5p. The expression of circRNA_004662 is elevated in UC patients, which destroys the intestinal epithelial barrier and may be involved in the pathogenesis of the disease.

#### 3.3.2 CircRNA and primary biliary cholangitis (PBC)

PBC is a rare autoimmune cholestatic liver disease that can be identified by persistent lymphocyte infiltration leading to the progressive deterioration of small bile ducts in the liver. This can consequently progress into liver fibrosis. The pathogenesis of PBC is multifaceted, involving autoimmune, environmental, genetic, and other factors. However, the fundamental mechanism remains elusive.^70^ In a study by Zheng et al., circRNA expression profiles were assessed from plasma samples from 6 affected individuals and 6 healthy controls, with subsequent validation done through qRT‐PCR in a larger cohort comprising of 29 PBC patients and 30 healthy controls.^71^ The findings unveiled 18 up‐regulated and 4 down‐regulated circRNAs in individuals with PBC in comparison to the healthy controls. Specifically hsa_circ_402458 (*p* = .0033), hsa_circ_087631, and hsa_circ_406329 (*p* = .0185) were demonstrated to be up‐regulated in the PBC cohort, whereas hsa_circ_407176 (*p* = .0066) and hsa_circ_082319 were down‐regulated. Notably, hsa_circ_402458 displayed significantly higher expression in PBC patients without ursodeoxycholic acid (UDCA) treatment compared to those receiving UDCA (*p* = .0338). ROC analysis for hsa_circ_402458 in diagnosing PBC yielded an AUC of 0.710 (*p* = .005), making it the most promising candidate marker. CeRNA analysis suggests that hsa_circ_402458 might target hsa‐miR‐522‐3p and hsa‐miR‐943. MiR‐522‐3p has been implicated in abnormal inflammation resolution and is associated with chronic inflammatory conditions. Furthermore, miR‐522‐3p directly binds to the 3′‐untranslated region (3′‐UTR) of secreted frizzled‐related protein 2 (SFRP2), a recognized Wnt signaling inhibitor, suggesting a potential role of circRNA dysregulation in PBC pathogenesis.^69^ Additionally, miR‐943 plays a role in DNA double‐strand break repair via the transforming growth factor‐β (TGF‐β) signaling pathway, where abnormal TGF‐β1 signaling has been linked to PBC development in mouse models.^72^ It suggests that hsa_circ_402458 could function as a miRNA sponge, potentially contributing to PBC by regulating inflammatory signaling pathways. Nevertheless, the precise functions of these miRNA–circRNA interactions in the pathogenesis require additional exploration. The abnormal expression of circRNA in the plasma of PBC patients may serve as a diagnostic biomarker for its diagnosis, and the dysregulation of circRNA may be involved in its development by regulating inflammatory signaling pathways.

### 3.4 CircRNA is associated with autoimmune diseases of the central nervous system

#### 3.4.1 CircRNA and multiple sclerosis (MS)

MS stands out as the primary non‐traumatic debilitating disease that impacts the younger demographic.^73^ It is a chronic autoimmune disorder affecting the CNS and is characterized by severe demyelination and continuous nerve damage.^74^ MS incidence is on the rise worldwide.^75^ The underlying cause of the disease remains uncertain.^76^ Relevant research used microarray chips to conduct differential expression profiling of circRNAs in four untreated relapsing remitting multiple sclerosis (RR‐MS) patients in the remission period and four HCs.^77^ In comparison to the control, hsa_circs of _0024892, _0000517, _0000519, _0000520, _001846, _0005402, _0000518, _000167, _0035560, _0005402, _101539, and _101541 expressions in individuals with MS were decreased. After qPCR validation, research findings also indicated that circ_0005402 and _0035560 exhibited decreased expression levels in individuals with MS, suggesting their potential utility as biomarkers for this condition. These circRNA expression profiles provide a new potential biomarker for the diagnosis and treatment of MS.

### 3.5 CircRNA is associated with skin‐related autoimmune diseases

#### 3.5.1 CircRNA and psoriasis

Psoriasis, an inflammatory skin disorder associated with various other conditions, affects over 60 million individuals globally, encompassing both adults and children.^78^ Initially perceived with having a primary root in epidermal keratinocytes, it is now recognized as being a prevalent immune‐mediated disorder, with significant contribution from factors such as TNFα, and cells like dendritic and T cells.^79^ While various medications and biological therapies are employed for treating moderate to severe psoriasis, these interventions offer symptom relief rather than a cure, and ongoing research is attempting to unravel the complexities of psoriasis's pathogenesis.^80^ CircRNAs, recognized for their complex regulatory functions in immunity, inflammation, and cell proliferation, have emerged as significant contributors to psoriasis development, offering new opportunities for therapeutic exploration.^71^, ^81^ CircRNAs are pivotal modulatory factors in psoriasis progression and are potential new targets of psoriasis treatment. Meng et al. demonstrated hsa_circ_0061012 was up‐regulated in psoriatic lesions compared with normal healthy skin tissues.^82^ Analyzing the top 5 microRNA response elements (MREs), hsa_circ_0061012, has‐miR‐7157‐5p, _4769‐3p, _6817‐5p, _4310, and _6882‐3p were identified through GO analysis, unveiling that the biological functions of these circular RNAs were linked to psoriasis. This underscores the potential of hsa_circ_0061012 as a biomarker for psoriasis, paving the way for a deeper understanding of circRNA function in psoriasis. In a study, Yang et al. identified 4 up‐regulated and 4 down‐regulated circRNAs in psoriatic regulatory T cells (Tregs) through circRNA microarray analysis.^83^ The notable elevation of circ_0003738 in psoriatic Tregs was validated through RT‐PCR. Notably, the knockout of circ_0003738 in psoriatic Tregs restored their inhibitory function to suppress pro‐inflammatory cytokines secretion, including interleukin‐17A (IL‐17A) and IFN‐γ. At a mechanistic level, circ_0003738 was found to interact with miR‐562, thereby alleviating the suppression of the target gene IL‐17 receptor A (IL‐17 RA) and consequently promoting IL‐17A signaling. MiR‐490‐5p affects Treg cell function by targeting IFNGR2, a subunit of the interferon‐γ (IFN‐γ) receptor, which plays a crucial role in immune regulation. In Treg cells, the regulation of IFNGR2 by miR‐490‐5p alters the response of these cells to IFN‐γ signaling. Impaired Treg cell function diminishes their suppressive effect on autoreactive T cells and other immune cells. In the pathogenesis of psoriasis, this immune imbalance exacerbates the inflammatory response leading to the commonly known symptoms of psoriasis.^83^

Circ_0003738 serves as a regulatory sponge for miR‐490‐5p, relieving the suppression of the target gene IFNGR2 and consequently enhancing IFN‐γ signaling in psoriatic Tregs. This study emphasized the role of up‐regulated circ_0003738 in attenuating the suppressive effect of psoriatic Tregs by sponging miR‐562/IL17RA and miR‐490‐5p/IFNGR2 axes, revealing possible novel therapeutic targets for psoriasis treating. Therefore, circRNAs are pivotal factors in psoriasis progression and could be new therapeutic targets.

## 4 CONCLUSION AND PERSPECTIVE

In summary, circRNA molecules exhibit distinct characteristics such as high conservation, abundant expression, stable structure, tissue specificity, and developmental specificity. The continuous progress in high‐throughput and microarray technologies have shed light on the intricate role of circRNAs in the complex pathophysiological regulatory processes of AIDs. Their dysregulated expression has been widely acknowledged by research findings as a contributor to varieties of autoimmune disorders, offering a novel research perspective for AID diagnosis and treatment. CircRNAs stand out as promising biomarkers for AID, offering valuable insights for disease diagnosis and prognosis prediction. Some circRNAs demonstrate abnormal expression in the blood or tissues of autoimmune disease patients, with their levels often correlating with the severity of the disease. Moreover, circRNAs present as potential therapeutic targets, opening up avenues for novel treatment approaches. For example, a circRNA vaccine against the Severe Acute Respiratory Syndrome Coronavirus 2 (SARS‐CoV‐2) variant was developed and used during the recent pandemic. In addition, several biotechnology companies are developing circRNA‐based therapies to treat certain diseases.^8^, ^84^ One such example, circOSBPL10, may serve as a prognostic factor and a potential biomarker for gastric cancer.^85^ Wu's team discovered that circTULP4 expression was diminished in Min6 cells under lipotoxic conditions.^86^ They found that circTULP4 could interact with miR‐7222‐3p to inhibit the expression of the cholesterol esterification‐related gene sterol O‐acyltransferase (SOAT1). Furthermore, the accumulation of SOAT1 in pancreatic islet β cells activated the expression of cell cycle protein D1, which promotes cell cycle progression and regulates β‐cell proliferation. Therefore, the up‐regulation of circTULP4 may be a promising therapeutic strategy for T2DM.

But there are still some shortcomings in this field: First, the existing clinical observations and experimental studies regarding the role of circRNA in AIDs are not comprehensive, and the understanding and conclusions drawn are still a bit superficial so far. Second, many reports focus on a particular circRNA, and the knowledge on the upstream regulatory mechanisms of each circRNA and their related biological function in AIDs remains unknown. Third, even with modern technology, current research equipment is still limited, and the research methods are not mature enough. Various technologies, such as RNA‐SEQ and RNA pull‐down technology, have been applied to the research of circRNA, and it is expected that more functions will eventually be discovered and various diseases can be better understood at the genetic level. This is necessary to accurately identify the circRNAs with associated regulatory effects and to analyze their underlying mechanism. Fourth, systematic large‐scale human sample studies are still scarce in this field. Fifth, the small sample size used to construct circRNA expression profiles may lead to low statistical power. Future large‐scale human studies and advanced bioinformatics tools are necessary to fully elucidate the role of circRNAs in AIDs. Future research efforts should also comprehensively investigate the functions of circRNAs in both AID patients and healthy individuals. This comprehensive approach should encompass various regulatory RNA species, including but not limited to circRNAs, miRNAs, and lncRNAs, and mRNAs. Sophisticated circRNA data analysis and bioinformatics algorithms will be crucial for deciphering these complex regulatory networks. Comprehending the functions of circRNAs in healthy and diseased patients and exploring their potential for diagnostic, prognostic, and therapeutic purposes, are crucial avenues for future research. Conducting large‐scale, multicenter studies are imperative to address key questions and topics such as the identification of biomarkers with increased specificity, sensitivity, diagnostic, and prognostic predictive abilities. Conduct large‐scale clinical validation to assess the performance of circular RNA (circRNA) as a biomarker by collecting clinical samples and conducting validation studies. CircRNA is utilized in conjunction with other biomarkers, including co‐testing and multi‐omics analysis, to enhance the reliability and utility of circRNA as a biomarker. Examining potential interactions between circRNAs and downstream signaling pathways and assessing the effects of interventions on circRNA expression and subsequent pathways requires rigorous exploration through larger sample sizes and multicenter studies. This proposal promises to enhance our understanding of autoimmune diseases by exploring more efficient diagnostic and therapeutic approaches via a new perspective of circular RNA.

## AUTHOR CONTRIBUTIONS

X.L. designed and wrote the draft and prepared pictures and tables; P.W., S.Q., J.A., and J.W. extensively edited the manuscript; Z.D. and J.Z. conceptualized and edited the manuscript.

## FUNDING INFORMATION

This study was financially supported by the Science and Technology Department of Shanxi Province (2022KXJ‐019): Scientist + engineer team construction for Research and development and application of Integrated Chinese and Western Medicine in diagnosis and treatment of difficult thyroid diseases, China; Shanxi Central Administration Bureau (2022‐SLRH‐LG‐005), “Double Chain Integration” Research and Innovation Team of Chinese and Western Integrated Thyroid Disease Diagnosis and Treatment, China; and the Science and Technology Department of Shanxi Province (2023‐ZLSF‐56), Joint Research and Development of hospital preparations for Hashimoto thyroiditis, Exploration of therapeutic mechanism and Formulation of TCM diagnosis and treatment Plan, China.

## DISCLOSURES

The authors declare no competing interest.

## Supporting information


**Figure S1.**.

**Figure S2.**.

**Figure S3.**.

**Figure S4.**.

**Figure S5.**.

## Data Availability

Data sharing is not applicable to this article as no datasets were generated or analyzed during the current study.

## References

[1] Conrad N , Misra S , Verbakel JY , et al. Incidence, prevalence, and co‐occurrence of autoimmune disorders over time and by age, sex, and socioeconomic status: a population‐based cohort study of 22 million individuals in the UK. Lancet. 2023;401(10391):1878‐1890. 37156255 10.1016/S0140-6736(23)00457-9

[2] Memczak S , Jens M , Elefsinioti A , et al. Circular RNAs are a large class of animal RNAs with regulatory potency. Nature. 2013;495(7441):333‐338. 23446348 10.1038/nature11928

[3] Li Z , Huang C , Bao C , et al. Exon‐intron circular RNAs regulate transcription in the nucleus. Nat Struct Mol Biol. 2015;22(3):256‐264. 25664725 10.1038/nsmb.2959

[4] Hansen TB , Jensen TI , Clausen BH , et al. Natural RNA circles function as efficient microRNA sponges. Nature. 2013;495(7441):384‐388. 23446346 10.1038/nature11993

[5] Pamudurti NR , Bartok O , Jens M , et al. Translation of CircRNAs. Mol Cell. 2017;66(1):9‐21.e27. 28344080 10.1016/j.molcel.2017.02.021 PMC5387669

[6] Sanger HL , Klotz G , Riesner D , Gross HJ , Kleinschmidt AK . Viroids are single‐stranded covalently closed circular RNA molecules existing as highly base‐paired rod‐like structures. Proc Natl Acad Sci USA. 1976;73(11):3852‐3856. 1069269 10.1073/pnas.73.11.3852 PMC431239

[7] Patop IL , Wüst S , Kadener S . Past, present, and future of circRNAs. EMBO J. 2019;38(16):e100836. 31343080 10.15252/embj.2018100836 PMC6694216

[8] Misir S , Wu N , Yang BB . Specific expression and functions of circular RNAs. Cell Death Differ. 2022;29(3):481‐491. 35169296 10.1038/s41418-022-00948-7 PMC8901656

[9] Zhang Y , Tan Y , Yuan J , et al. circLIFR‐007 reduces liver metastasis via promoting hnRNPA1 nuclear export and YAP phosphorylation in breast cancer. Cancer Lett. 2024;592:216907. 38685451 10.1016/j.canlet.2024.216907

[10] Han B , Chao J , Yao H . Circular RNA and its mechanisms in disease: from the bench to the clinic. Pharmacol Ther. 2018;187:31‐44. 29406246 10.1016/j.pharmthera.2018.01.010

[11] Liu X , Wang X , Li J , et al. Identification of mecciRNAs and their roles in the mitochondrial entry of proteins. Sci China Life Sci. 2020;63(10):1429‐1449. 32048164 10.1007/s11427-020-1631-9

[12] Huang C , Liang D , Tatomer DC , Wilusz JE . A length‐dependent evolutionarily conserved pathway controls nuclear export of circular RNAs. Genes Dev. 2018;32(9–10):639‐644. 29773557 10.1101/gad.314856.118 PMC6004072

[13] Tao X , Shao Y , Yan J , et al. Biological roles and potential clinical values of circular RNAs in gastrointestinal malignancies. Cancer Biol Med. 2021;18(2):437‐457. 33710802 10.20892/j.issn.2095-3941.2020.0348 PMC8185857

[14] Chen LL . The expanding regulatory mechanisms and cellular functions of circular RNAs. Nat Rev Mol Cell Biol. 2020;21(8):475‐490. 32366901 10.1038/s41580-020-0243-y

[15] Khan FA , Nsengimana B , Khan NH , et al. Chimeric peptides/proteins encoded by circRNA: an update on mechanisms and functions in human cancers. Front Oncol. 2022;12:781270. 35223470 10.3389/fonc.2022.781270 PMC8874284

[16] Salzman J , Chen RE , Olsen MN , Wang PL , Brown PO . Cell‐type specific features of circular RNA expression. PLoS Genet. 2013;9(9):e1003777. 24039610 10.1371/journal.pgen.1003777 PMC3764148

[17] Li X , Yang L , Chen LL . The biogenesis, functions, and challenges of circular RNAs. Mol Cell. 2018;71(3):428‐442. 30057200 10.1016/j.molcel.2018.06.034

[18] Xiao MS , Ai Y , Wilusz JE . Biogenesis and functions of circular RNAs come into focus. Trends Cell Biol. 2020;30(3):226‐240. 31973951 10.1016/j.tcb.2019.12.004 PMC7069689

[19] Liu CX , Chen LL . Circular RNAs: characterization, cellular roles, and applications. Cell. 2022;185(12):2016‐2034. 35584701 10.1016/j.cell.2022.04.021

[20] Steinman L . Autoimmune disease. Sci Am. 1993;269(3):106‐114. 10.1038/scientificamerican0993-106 8211084

[21] Sthoeger Z , Zinger H , Sharabi A , Asher I , Mozes E . The tolerogenic peptide, hCDR1, down‐regulates the expression of interferon‐α in murine and human systemic lupus erythematosus. PLoS ONE. 2013;8(3):e60394. 23555966 10.1371/journal.pone.0060394 PMC3610660

[22] Sela U , Dayan M , Hershkoviz R , Lider O , Mozes E . A peptide that ameliorates lupus up‐regulates the diminished expression of early growth response factors 2 and 3. J Immunol. 2008;180(3):1584‐1591. 18209054 10.4049/jimmunol.180.3.1584

[23] Agirre X , Meydan C , Jiang Y , et al. Long non‐coding RNAs discriminate the stages and gene regulatory states of human humoral immune response. Nat Commun. 2019;10(1):821. 30778059 10.1038/s41467-019-08679-z PMC6379396

[24] Song M , Gao J , Yan T , et al. Hsa_circ_0000652 aggravates inflammation by activation of macrophages and enhancement of OX40/OX40L interaction in ankylosing spondylitis. Front Cell Dev Biol. 2021;9:737599. 34977002 10.3389/fcell.2021.737599 PMC8716807

[25] Jiang Z , Huang L , Chen L , et al. Circular RNA profile in Graves' disease and potential function of hsa_circ_0090364. Endocr Connect. 2022;11(11):e220030. 36018563 10.1530/EC-22-0030 PMC9578071

[26] Zhang Z , Luo S , Xiao Z , et al. Hsa_circRNA_405498 and hsa_circRNA_100033 serve as potential biomarkers for differential diagnosis of type 1 diabetes. J Clin Endocrinol Metab. 2024;109(6):1464‐1473. 38157408 10.1210/clinem/dgad761

[27] Liu CX , Li X , Nan F , et al. Structure and degradation of circular RNAs regulate PKR activation in innate immunity. Cell. 2019;177(4):865‐880.e821. 31031002 10.1016/j.cell.2019.03.046

[28] Zheng ZM . Correction to: circular RNAs and RNase L in PKR activation and virus infection. Cell Biosci. 2019;9:58. 31346407 10.1186/s13578-019-0320-0 PMC6636110

[29] Launer‐Felty K , Cole JL . Domain interactions in adenovirus VAI RNA mediate high‐affinity PKR binding. J Mol Biol. 2014;426(6):1285‐1295. 24394721 10.1016/j.jmb.2013.12.019 PMC3961479

[30] Li X , Liu CX , Xue W , et al. Coordinated circRNA biogenesis and function with NF90/NF110 in viral infection. Mol Cell. 2017;67(2):214‐227.e217. 28625552 10.1016/j.molcel.2017.05.023

[31] Wu TH , Shi L , Adrian J , et al. NF90/ILF3 is a transcription factor that promotes proliferation over differentiation by hierarchical regulation in K562 erythroleukemia cells. PLoS ONE. 2018;13(3):e0193126. 29590119 10.1371/journal.pone.0193126 PMC5873942

[32] Wang YH , Yu XH , Luo SS , Han H . Comprehensive circular RNA profiling reveals that circular RNA100783 is involved in chronic CD28‐associated CD8(+)T cell ageing. Immun Ageing. 2015;12:17. 26451160 10.1186/s12979-015-0042-z PMC4597608

[33] Kiriakidou M , Ching CL . Systemic lupus erythematosus. Ann Intern Med. 2020;172(11):ITC81‐ITC96. 32479157 10.7326/AITC202006020

[34] Tsokos GC . Systemic lupus erythematosus. N Engl J Med. 2011;365(22):2110‐2121. 22129255 10.1056/NEJMra1100359

[35] Zheng F , Yu X , Tang D , et al. The identification of circular RNAs from peripheral blood mononuclear cells in systemic lupus erythematosus. BMC Med Genet. 2021;14(1):70. 10.1186/s12920-021-00919-w PMC7941743 33750387

[36] Luo Q , Zhang L , Fang L , et al. Circular RNAs hsa_circ_0000479 in peripheral blood mononuclear cells as novel biomarkers for systemic lupus erythematosus. Autoimmunity. 2020;53(3):167‐176. 32093518 10.1080/08916934.2020.1728529

[37] Luo Q , Zhang L , Li X , et al. Identification of circular RNAs hsa_circ_0044235 and hsa_circ_0068367 as novel biomarkers for systemic lupus erythematosus. Int J Mol Med. 2019;44(4):1462‐1472. 31432107 10.3892/ijmm.2019.4302 PMC6713423

[38] Li S , Zhang J , Tan X , et al. Microarray expression profile of circular RNAs and mRNAs in children with systemic lupus erythematosus. Clin Rheumatol. 2019;38(5):1339‐1350. 30628013 10.1007/s10067-018-4392-8

[39] Zhang C , Wang X , Chen Y , Wu Z , Zhang C , Shi W . The down‐regulation of hsa_circ_0012919, the sponge for miR‐125a‐3p, contributes to DNA methylation of CD11a and CD70 in CD4(+) T cells of systemic lupus erythematous. Clin Sci (Lond). 2018;132(21):2285‐2298. 30237316 10.1042/CS20180403

[40] Ouyang Q , Huang Q , Jiang Z , Zhao J , Shi GP , Yang M . Using plasma circRNA_002453 as a novel biomarker in the diagnosis of lupus nephritis. Mol Immunol. 2018;101:531‐538. 30172209 10.1016/j.molimm.2018.07.029

[41] Zhang C , Huang J , Chen Y , Shi W . Low expression and clinical value of hsa_circ_0049224 and has_circ_0049220 in systemic lupus erythematous patients. Med Sci Monit. 2018;24:1930‐1935. 29606700 10.12659/MSM.906507 PMC5898388

[42] Li H , Li K , Lai W , et al. Comprehensive circular RNA profiles in plasma reveals that circular RNAs can be used as novel biomarkers for systemic lupus erythematosus. Clin Chim Acta. 2018;480:17‐25. 29360436 10.1016/j.cca.2018.01.026

[43] Smolen JS , Aletaha D , McInnes IB . Rheumatoid arthritis. Lancet. 2016;388(10055):2023‐2038. 27156434 10.1016/S0140-6736(16)30173-8

[44] Yang J , Cheng M , Gu B , Wang J , Yan S , Xu D . CircRNA_09505 aggravates inflammation and joint damage in collagen‐induced arthritis mice via miR‐6089/AKT1/NF‐κB axis. Cell Death Dis. 2020;11(10):833. 33028811 10.1038/s41419-020-03038-z PMC7542153

[45] Wen J , Liu J , Zhang P , et al. RNA‐seq reveals the circular RNA and miRNA expression profile of peripheral blood mononuclear cells in patients with rheumatoid arthritis. Biosci Rep. 2020;40(4):BSR20193160. 32191279 10.1042/BSR20193160 PMC7133114

[46] Luo Q , Zeng L , Zeng L , et al. Expression and clinical significance of circular RNAs hsa_circ_0000175 and hsa_circ_0008410 in peripheral blood mononuclear cells from patients with rheumatoid arthritis. Int J Mol Med. 2020;45(4):1203‐1212. 32124964 10.3892/ijmm.2020.4498

[47] Luo Q , Liu J , Fu B , et al. Circular RNAs Hsa_circ_0002715 and Hsa_circ_0035197 in peripheral blood are novel potential biomarkers for new‐onset rheumatoid arthritis. Dis Markers. 2019;2019:2073139. 31772684 10.1155/2019/2073139 PMC6855002

[48] Yang X , Li J , Wu Y , Ni B , Zhang B . Aberrant dysregulated circular RNAs in the peripheral blood mononuclear cells of patients with rheumatoid arthritis revealed by RNA sequencing: novel diagnostic markers for RA. Scand J Clin Lab Invest. 2019;79(8):551‐559. 31596149 10.1080/00365513.2019.1674004

[49] Li B , Li N , Zhang L , et al. Hsa_circ_0001859 regulates ATF2 expression by functioning as an MiR‐204/211 sponge in human rheumatoid arthritis. J Immunol Res. 2018;2018:9412387. 29577053 10.1155/2018/9412387 PMC5822876

[50] Ouyang Q , Wu J , Jiang Z , et al. Microarray expression profile of circular RNAs in peripheral blood mononuclear cells from rheumatoid arthritis patients. Cell Physiol Biochem. 2017;42(2):651‐659. 28618429 10.1159/000477883

[51] Constantopoulos SH , Tsianos EV , Moutsopoulos HM . Pulmonary and gastrointestinal manifestations of Sjögren's syndrome. Rheum Dis Clin N Am. 1992;18(3):617‐635. 1496165

[52] Li F , Liu Z , Zhang B , et al. Circular RNA sequencing indicates circ‐IQGAP2 and circ‐ZC3H6 as noninvasive biomarkers of primary Sjögren's syndrome. Rheumatology. 2020;59(9):2603‐2615. 32250392 10.1093/rheumatology/keaa163

[53] Su LC , Xu WD , Liu XY , Fu L , Huang AF . Altered expression of circular RNA in primary Sjögren's syndrome. Clin Rheumatol. 2019;38(12):3425‐3433. 31420809 10.1007/s10067-019-04728-6

[54] Kobayashi T , Tomofuji T , Machida T , et al. Expression of salivary miR‐203a‐3p was related with oral health‐related quality of life in healthy volunteers. Int J Mol Sci. 2017;18(6):1263. 28608821 10.3390/ijms18061263 PMC5486085

[55] Yang Z , Wang J , Pan Z , Zhang Y . miR‐143‐3p regulates cell proliferation and apoptosis by targeting IGF1R and IGFBP5 and regulating the Ras/p38 MAPK signaling pathway in rheumatoid arthritis. Exp Ther Med. 2018;15(4):3781‐3790. 29581736 10.3892/etm.2018.5907 PMC5863597

[56] Ralli M , Angeletti D , Fiore M , et al. Hashimoto's thyroiditis: an update on pathogenic mechanisms, diagnostic protocols, therapeutic strategies, and potential malignant transformation. Autoimmun Rev. 2020;19(10):102649. 32805423 10.1016/j.autrev.2020.102649

[57] Duntas LH , Hiromatsu Y , Amino N . Centennial of the description of hashimoto's thyroiditis: two thought‐provoking events. Thyroid. 2013;23(6):643‐645. 23544732 10.1089/thy.2012.0627

[58] Delemer B , Aubert JP , Nys P , Landron F , Bouée S . An observational study of the initial management of hypothyroidism in France: the ORCHIDÉE study. Eur J Endocrinol. 2012;167(6):817‐823. 23034782 10.1530/EJE-11-1041 PMC3494865

[59] Xiong S , Peng H , Ding X , et al. Circular RNA expression profiling and the potential role of hsa_circ_0089172 in Hashimoto's thyroiditis via sponging miR125a‐3p. Mol Ther Nucleic Acids. 2019;17:38‐48. 31207490 10.1016/j.omtn.2019.05.004 PMC6579753

[60] Bluestone JA , Buckner JH , Herold KC . Immunotherapy: building a bridge to a cure for type 1 diabetes. Science. 2021;373(6554):510‐516. 34326232 10.1126/science.abh1654

[61] Sun H , Saeedi P , Karuranga S , et al. IDF diabetes atlas: global, regional and country‐level diabetes prevalence estimates for 2021 and projections for 2045. Diabetes Res Clin Pract. 2022;183:109119. 34879977 10.1016/j.diabres.2021.109119 PMC11057359

[62] Kaur S , Mirza AH , Pociot F . Cell type‐selective expression of circular RNAs in human pancreatic islets. Non‐coding RNA. 2018;4(4):38. 30486482 10.3390/ncrna4040038 PMC6316812

[63] Zhang C , Han X , Yang L , et al. Circular RNA circPPM1F modulates M1 macrophage activation and pancreatic islet inflammation in type 1 diabetes mellitus. Theranostics. 2020;10(24):10908‐10924. 33042261 10.7150/thno.48264 PMC7532688

[64] Yang L , Han X , Zhang C , et al. Hsa_circ_0060450 negatively regulates type I interferon‐induced inflammation by serving as miR‐199a‐5p sponge in type 1 diabetes mellitus. Front Immunol. 2020;11:576903. 33133095 10.3389/fimmu.2020.576903 PMC7550460

[65] Wright EK , Ding NS , Niewiadomski O . Management of inflammatory bowel disease. Med J Aust. 2018;209(7):318‐323. 30257634 10.5694/mja17.01001

[66] Veauthier B , Hornecker JR . Crohn's disease: diagnosis and management. Am Fam Physician. 2018;98(11):661‐669. 30485038

[67] Ye YL , Yin J , Hu T , Zhang LP , Wu LY , Pang Z . Increased circulating circular RNA_103516 is a novel biomarker for inflammatory bowel disease in adult patients. World J Gastroenterol. 2019;25(41):6273‐6288. 31749597 10.3748/wjg.v25.i41.6273 PMC6848015

[68] Yin J , Hu T , Xu L , et al. Circular RNA expression profile in peripheral blood mononuclear cells from Crohn disease patients. Medicine. 2019;98(26):e16072. 31261517 10.1097/MD.0000000000016072 PMC6617429

[69] Shi J , Ma H , Wang H , et al. Overexpression of LINC00261 inhibits non‐small cell lung cancer cells progression by interacting with miR‐522‐3p and suppressing Wnt signaling. J Cell Biochem. 2019;120(10):18378‐18387. 31190356 10.1002/jcb.29149

[70] Levy C , Manns M , Hirschfield G . New treatment paradigms in primary biliary cholangitis. Clin Gastroenterol Hepatol. 2023;21(8):2076‐2087. 36809835 10.1016/j.cgh.2023.02.005

[71] Zheng J , Li Z , Wang T , Zhao Y , Wang Y . Microarray expression profile of circular RNAs in plasma from primary biliary cholangitis patients. Cell Physiol Biochem. 2017;44(4):1271‐1281. 29183005 10.1159/000485487

[72] Liu B , Zhang X , Zhang FC , Zong JB , Zhang W , Zhao Y . Aberrant TGF‐β1 signaling contributes to the development of primary biliary cirrhosis in murine model. World J Gastroenterol. 2013;19(35):5828‐5836. 24124327 10.3748/wjg.v19.i35.5828 PMC3792337

[73] Kobelt G , Thompson A , Berg J , Gannedahl M , Eriksson J . New insights into the burden and costs of multiple sclerosis in Europe. Mult Scler. 2017;23(8):1123‐1136. 28273775 10.1177/1352458517694432 PMC5476197

[74] Adams BD , Parsons C , Walker L , Zhang WC , Slack FJ . Targeting noncoding RNAs in disease. J Clin Invest. 2017;127(3):761‐771. 28248199 10.1172/JCI84424 PMC5330746

[75] Browne P , Chandraratna D , Angood C , et al. Atlas of multiple sclerosis 2013: a growing global problem with widespread inequity. Neurology. 2014;83(11):1022‐1024. 25200713 10.1212/WNL.0000000000000768 PMC4162299

[76] Dobson R , Giovannoni G . Multiple sclerosis—a review. Eur J Neurol. 2019;26(1):27‐40. 30300457 10.1111/ene.13819

[77] Iparraguirre L , Muñoz‐Culla M , Prada‐Luengo I , Castillo‐Triviño T , Olascoaga J , Otaegui D . Circular RNA profiling reveals that circular RNAs from ANXA2 can be used as new biomarkers for multiple sclerosis. Hum Mol Genet. 2017;26(18):3564‐3572. 28651352 10.1093/hmg/ddx243

[78] Michalek IM , Loring B , John SM . A systematic review of worldwide epidemiology of psoriasis. J Eur Acad Dermatol Venereol. 2017;31(2):205‐212. 27573025 10.1111/jdv.13854

[79] Griffiths CE , Barker JN . Pathogenesis and clinical features of psoriasis. Lancet. 2007;370(9583):263‐271. 17658397 10.1016/S0140-6736(07)61128-3

[80] Ovejero‐Benito MC , Muñoz‐Aceituno E , Reolid A , Saiz‐Rodríguez M , Abad‐Santos F , Daudén E . Pharmacogenetics and pharmacogenomics in moderate‐to‐severe psoriasis. Am J Clin Dermatol. 2018;19(2):209‐222. 28921458 10.1007/s40257-017-0322-9

[81] Zhou Z , Du D , Chen A , Zhu L . Circular RNA expression profile of articular chondrocytes in an IL‐1β‐induced mouse model of osteoarthritis. Gene. 2018;644:20‐26. 29247798 10.1016/j.gene.2017.12.020

[82] Qiao M , Ding J , Yan J , Li R , Jiao J , Sun Q . Circular RNA expression profile and analysis of their potential function in psoriasis. Cell Physiol Biochem. 2018;50(1):15‐27. 30278433 10.1159/000493952

[83] Yang L , Zhang C , Bai X , Xiao C , Dang E , Wang G . hsa_circ_0003738 inhibits the suppressive function of Tregs by targeting miR‐562/IL‐17A and miR‐490‐5p/IFN‐γ signaling pathway. Mol Ther Nucleic Acids. 2020;21:1111‐1119. 32871353 10.1016/j.omtn.2020.08.001 PMC7475646

[84] Wu N , Qadir J , Yang BB . CircRNA perspective: new strategies for RNA therapy. Trends Mol Med. 2022;28(4):343‐344. 35232670 10.1016/j.molmed.2022.02.002

[85] Wang S , Zhang X , Li Z , et al. Circular RNA profile identifies circOSBPL10 as an oncogenic factor and prognostic marker in gastric cancer. Oncogene. 2019;38(44):6985‐7001. 31409903 10.1038/s41388-019-0933-0

[86] Wu L , Xiong L , Li J , et al. Circ‐Tulp4 promotes β‐cell adaptation to lipotoxicity by regulating soat1 expression. J Mol Endocrinol. 2020;65(4):149‐161. 33064661 10.1530/JME-20-0079 PMC7576671

